# Ketone body levels and its associations with cardiac markers following an acute myocardial infarction: a post hoc analysis of the EMMY trial

**DOI:** 10.1186/s12933-024-02221-2

**Published:** 2024-04-27

**Authors:** Faisal Aziz, Norbert J. Tripolt, Peter N. Pferschy, Hubert Scharnagl, Mahmoud Abdellatif, Abderrahim Oulhaj, Martin Benedikt, Ewald Kolesnik, Dirk von Lewinski, Harald Sourij

**Affiliations:** 1 Interdisciplinary Metabolic Medicine Trials Unit, Medical University of Graz, Graz, Austria; 2 Clinical Institute for Chemical and Medical Laboratory Analysis, Medical University of Graz, Graz, Austria; 3 Division of Cardiology, Medical University of Graz, Graz, Austria; 4 Department of Public Health and Epidemiology, College of Medicine and Health Sciences, Khalifa University of Sciences and Technology, Abu Dhabi, United Arab Emirates; 5 Biotechnology Center, Khalifa University of Sciences and Technology, Abu Dhabi, United Arab Emirates; 6 Working Group Myocardial Energetics and Metabolism, Medical University of Graz, Graz, Austria; 7 Division of Endocrinology and Diabetology, Medical University of Graz, Graz, Austria

**Keywords:** SGLT2 inhibitor, Empagliflozin, Ketone body, Beta-hydroxybutyrate, 3-βOHB, Clinical Trial

## Abstract

**Background:**

Sodium-glucose co-transporter 2 inhibitors (SGLT2i) have been suggested to exert cardioprotective effects in patients with heart failure, possibly by improving the metabolism of ketone bodies in the myocardium.

**Methods:**

This post hoc analysis of the EMMY trial investigated the changes in serum β-hydroxybutyrate (3-βOHB) levels after acute myocardial infarction (AMI) in response to 26-week of Empagliflozin therapy compared to the usual post-MI treatment. In addition, the association of baseline and repeated measurements of 3-βOHB with cardiac parameters and the interaction effects of Empagliflozin were investigated. Cardiac parameters included N-terminal pro-B-type natriuretic peptide (NT-proBNP), left ventricular ejection fraction (LVEF), left ventricle end-systolic volume (LVESV), left ventricle end-diastolic volume (LVEDV), and left ventricular filling pressure (E/é ratio).

**Results:**

The mean 3-βOHB levels increased from baseline (46.2 ± 3.0 vs. 51.7 ± 2.7) to 6 weeks (48.8 ± 2.2 vs. 42.0 ± 2.3) and 26 weeks (49.3 ± 2.2 vs. 35.8 ± 1.9) in the Empagliflozin group compared to a consistent decline in placebo over 26 weeks (p_interaction_ < 0.001). Baseline and longitudinal measurements of 3-βOHB were not significantly associated with NT-proBNP and E/é ratio. Baseline 3-βOHB value was negatively associated with LVEF (coefficient: − 0.464, 95%CI − 0.863;− 0.065, p = 0.023), while an increase in its levels over time was positively associated with LVEF (0.595, 0.156;1.035, 0.008). The baseline 3-βOHB was positively associated with LVESV (1.409, 0.186;2.632, 0.024) and LVEDV (0.640, − 1.170;− 2.449, 0.488), while an increase in its levels over time was negatively associated with these cardiac parameters (LVESV: − 2.099, − 3.443;− 0.755, 0.002; LVEDV: − 2.406, − 4.341;− 0.472, 0.015). Empagliflozin therapy appears to modify the association between 3-βOHB, LVEF (p_interaction_ = 0.090), LVESV (p_interaction_ = 0.134), and LVEDV (p_interaction_ = 0.168), particularly at 26 weeks; however, the results were not statistically significant.

**Conclusion:**

This post hoc analysis showed that SGLT2i increased 3-βOHB levels after AMI compared to placebo. Higher baseline 3-βOHB levels were inversely associated with cardiac function at follow-up, whereas a sustained increase in 3-βOHB levels over time improved these markers. This highlights the importance of investigating ketone body metabolism in different post-MI phases. Although more pronounced effect of 3-βOHB on cardiac markers was observed in the SGLT2i group, further research is required to explore this interaction effect.

## Background

Ketone bodies including acetoacetate, acetone, and β-hydroxybutyrate (3-βOHB) are synthesized by ketogenesis in the liver and oxidized in various tissues via ketolytic pathways. Under physiological conditions, ketone bodies contribute to 10–20% of cardiac energy metabolism [1, 2], however, their metabolism increases substantially in hypertrophic and ischemic cardiac conditions to meet energy demands [3, 4]. This energy shift offers various clinical benefits to cardiac patients such as improvement in myocardial blood flow, cardiac function, cardiorenal protection, and energy efficiency [5].

Sodium-glucose co-transporter 2 inhibitors (SGLT2i) are well-known to exert cardioprotective effects in patients with heart failure and other clinical conditions by influencing various inflammatory, cardio-renal, and metabolic pathways [6–9]. Recent evidence from clinical trials and real-world data has also suggested various cardiometabolic and cardiovascular benefits of early administration of SGLT2i in patients with acute myocardial infarction (AMI) [10–15]. However, the exact mechanisms responsible for the cardioprotective effects of this drug class are still being explored.

Emerging experimental, metabolomic, and clinical data from heart failure and AMI models and humans suggest that SGLT2i have the potential to increase ketonemia and myocardial utilization of ketones by promoting lipolysis and ketogenesis as a result of lowering insulin-to-glucose ratio, which then attenuates oxidative stress and inflammatory process and improves mitochondrial function, adenosine triphosphate (ATP) production, cardiac metabolism, and cardiac function [16–20]. Therefore, SGLT2i-induced elevation in ketone bodies, particularly 3-βOHB, has been proposed to play a mediatory role in cardioprotective effects. However, clinical research exploring the influence of SGLT2i therapy on circulatory ketone bodies in acute and late post-MI phases is scarce. In addition, elevated ketone body levels following AMI have been found to be positively correlated with infarction severity and poor ejection fraction in AMI patients [21], while supplementation of ketones in animal models after AMI has been shown to improve cardiac metabolism, energetics, and remodeling [18, 22, 23]. Therefore, it is reasonable to assume that acute and follow-up levels, or particularly SGLT2i therapy-induced increase in 3-βOHB would impact differently on cardiac parameters.

Our recently published Empagliflozin in acute myocardial infarction (EMMY) trial demonstrated significant benefits of 26-week Empagliflozin therapy on cardiac biomarkers and functional as well as structural cardiac metrics in AMI patients [10]. In this post-hoc analysis, we investigated alterations in 3-βOHB levels in response to 26 weeks of Empagliflozin therapy compared to placebo in AMI patients enrolled in the EMMY trial. In addition, we examined the association of both baseline (measured within 72 h after AMI) and longitudinal measurements (follow-up) of 3-βOHB with the change in cardiac markers over 26 weeks and assessed whether the Empagliflozin therapy in the EMMY trial patients modified these associations.

## Methods

### EMMY trial

The EMMY trial investigated the effect of oral Empagliflozin treatment administered 10 mg once a day compared with routine post-MI treatment on various structural and functional cardiac parameters in 476 people who suffered from AMI. Treatment was commenced within 72 h after the percutaneous coronary intervention for the acute event. Relevant clinical, laboratory, and outcomes data were collected at baseline and follow-up visits at 6 weeks, 12 weeks, and 26 weeks. The detailed methodology and primary results of EMMY have been published [10, 24]. The primary outcome of the trial was the change in N-terminal pro b-type natriuretic peptide (NT-proBNP) and the secondary outcomes were changes in left-ventricular ejection fraction (LVEF), left-ventricular end-systolic (LVESV) volume, left-ventricular end-diastolic volume (LVEDV), and E/é ratio.

### Post-hoc analysis

This post-hoc analysis of EMMY used 3-βOHB, NT-proBNP, LVEF, LVESV, LVEDV, and E/é ratio measurements collected at baseline (within 72 h of AMI) and follow-up visits at 6 weeks and 26 weeks. Serum 3-βOHB was measured in OlympusAU680 automated analyzer using an enzymatic assay from Diasys Diagnostic Systems (Holzheim, Germany).

Clinical data included in this post-hoc analysis comprised age, sex, treatment groups, body mass index (BMI), and systolic and diastolic blood pressure measurements. Use of concomitant medication included angiotensin-converting enzyme inhibitors/angiotensin receptor blockers (ACEI/ARB), angiotensin receptor/neprilysin inhibitor (ARNI), Beta-blockers, mineralocorticoid receptor antagonist (MRA), diuretics, calcium channel blockers, and lipid-lowering medicines. Laboratory measurements included estimated glomerular filtration rate (eGFR), high sensitivity C-reactive protein (hsCRP), serum lipoproteins, and cardiac enzymes such as creatine kinase, creatine kinase muscle-brain (MB), and Troponin T.

### Statistical analysis

All data were analyzed in Stata version 18.0. Qualitative variables were summarized as frequencies and percentages and compared with the treatment group using Chi-square or Fischer Exact tests, as appropriate. Quantitative variables were summarized as the median and interquartile range (IQR) and compared with the treatment group using the Wilcoxon rank-sum test.

The linear mixed effect model (LMEM) was applied to analyze the mean change in log-transformed 3-βOHB over visits and compare the average treatment effect and treatment-visit interaction on 3-βOHB levels. In addition, the model was adjusted for baseline age, sex, diabetes status, and 3-βOHB levels. In the LMEM model, individual patients were fitted as a random effect, whereas visit, treatment, and other covariates were fitted as fixed effects. The results of LMEM were reported and presented as marginal means of 3-βOHB at each visit with corresponding 95% confidence intervals (CI) and p-values.

The LMEM was also used to assess the association of both baseline (tertiles and continuous log values) and longitudinal 3-βOHB measurements with cardiac markers. In the model with baseline 3-βOHB, treatment was added as an interaction term and the association was adjusted for age, sex, diabetes status, and baseline values of each cardiac marker. In the model with longitudinal 3-βOHB, the interaction terms of 3-βOHB with treatment and visit were added, and the associations were adjusted for visit, age, sex, and diabetes status. The results of associations were reported as regression coefficients with corresponding 95%CI and p-values. The interaction effect of Empagliflozin therapy on the association of both baseline and longitudinal 3-βOHB levels with cardiac markers was reported for the overall follow-up period (Table 3) as well as at each visit (Fig. 2).

### Ethical considerations

The EMMY trial was approved by the Ethics Committee of the Medical University of Graz, Austria (EK 29–179 ex 16/17; EudraCT 2016-004591-22) and the relevant authorities of each participating study center and was registered on ClinicalTrials.gov (NCT03087773). The trial adhered to all ethical and good clinical practice standards set by the Declaration of Helsinki and the International Conference on Harmonization for Good Clinical Practice (ICH CGP E6).

## Results

### Baseline characteristics

A total of 374 participants with available measurements of 3-βOHB at all visits were analyzed in this post-hoc analysis. The EMMY trial participants were similar in terms of demographic characteristics, laboratory parameters, and cardiac markers between the Empagliflozin and placebo groups at baseline (Table 1).

**Table 1:** Baseline characteristics of EMMY trial participants with available ketone body measurements

Variables	All	Empagliflozin	Placebo
All, n (%)	374 (100.0%)	189 (50.5%)	185 (49.5%)
Age (years), median (IQR)	57 (52–64)	57 (52–64)	57 (52–64)
Sex, n (%)			
Male	305 (81.55%)	158 (83.60%)	147 (79.46%)
Female	69 (18.45%)	31 (16.40%)	38 (20.54%)
BMI, kg/m^2^, median (IQR)	28 (25–30)	28 (25–30)	28 (25–30)
Systolic blood pressure (mmHg), median (IQR)	125 (117–131)	125 (115–131)	125 (118–131)
Diastolic blood pressure (mmHg), median (IQR)	78 (74–85)	78 (74–84)	78 (74–85)
Type 2 diabetes, n (%)	51 (13.64%)	24 (12.70%)	27 (14.59%)
Hypertension, n (%)	157 (41.98%)	73 (38.62%)	84 (45.41%)
Dyslipidemia, n (%)	103 (27.54%)	60 (31.75%)	43 (23.24%)
CAD, n (%)	32 (8.56%)	19 (10.05%)	13 (7.03%)
Coronary angiography vessel status, n (%)			
1-vessel disease	180 (48.13%)	82 (43.39%)	98 (52.97%)
2-vessel disease	123 (32.89%)	65 (34.39%)	58 (31.35%)
3-vessel disease	71 (18.98%)	42 (22.22%)	29 (15.68%)
History of NSTEMI, n (%)	16 (4.28%)	11 (5.82%)	5 (2.70%)
Treatment			
ACE-1/ARB, n (%)	361 (97.57%)	184 (97.87%)	177 (97.25%)
ARNI, n (%)	6 (1.60%)	1 (0.53%)	5 (2.70%)
Beta-blocker, n (%)	360 (96.26%)	179 (94.71%)	181 (97.84%)
MRA, n (%)	145 (38.77%)	70 (37.04%)	75 (40.54%)
Loop diuretic, n (%)	36 (9.63%)	20 (10.58%)	16 (8.65%)
Calcium channel blocker, n (%)	17 (4.55%)	8 (4.23%)	9 (4.86%)
Statin, n (%)	368 (98.40%)	185 (97.88%)	183 (98.92%)
Laboratory parameters			
eGFR (ml/min), median (IQR)	92 (78–101)	93 (78–101)	91 (78–101)
hsCRP (mg/dl), median (IQR)	7 (2–15)	6 (2–13)	7 (3–16)
Total cholesterol (mg/dL), median (IQR)	192 (165–223)	192 (165–225)	191 (166–221)
LDL-C, (mg/dL), median (IQR)	123 (96–150)	123 (98–152)	123 (92–146)
HDL-C (mg/dL), median (IQR)	43 (36–52)	43 (36–52)	43 (36–53)
Creatine kinase (U/l), median (IQR)	1648 (1200–2452)	1588 (1125–2467)	1670 (1257–2417)
Creatine kinase MB (U/l), median (IQR)	146 (84–230)	137 (80–227)	159 (84–237)
Troponin T (µg/l), median (IQR)	3042 (2041–4677)	3045 (2062–4628)	3039 (1996–4871)
NT-proBNP (pg/ml), median (IQR)	1365 (773–2192)	1272 (773–2127)	1421 (800–2192)
LVEF (%), median (IQR)	48 (43–54)	48 (43–53)	49 (44–55)
LVESV (ml), median (IQR)	61 (47–75)	62 (49–78)	60 (45–73)
LVEDV (ml), median (IQR)	119 (93–138)	121 (95–140)	114 (92–136)
E/é ratio, median (IQR)	9 (7–11)	9 (7–11)	9 (8–11)

*BMI* body mass index, *CAD* coronary artery disease, *ACE* angiotensin converting enzyme, *ARB* angiotensin receptor blocker, *ARNI* angiotensin receptor/neprilysin inhibitor, *MRA* mineralocorticoid receptor antagonist, *eGFR* estimated glomerular filtration rate, high sensitivity CRP: c-reactive protein, *LDL-C* low density lipoprotein-cholesterol, *HDL-C* high density lipoprotein-cholesterol, *NT-proBNP* N-terminal pro b-type natriuretic peptide**,** *LVEF* left ventricular ejection fraction, *LVESV* left ventricle end-systolic volume, *LVEDV* left ventricle end-diastolic volume, *E/é* left ventricular filling pressure

### 3-βOHB levels over visits

**Fig. 1:**
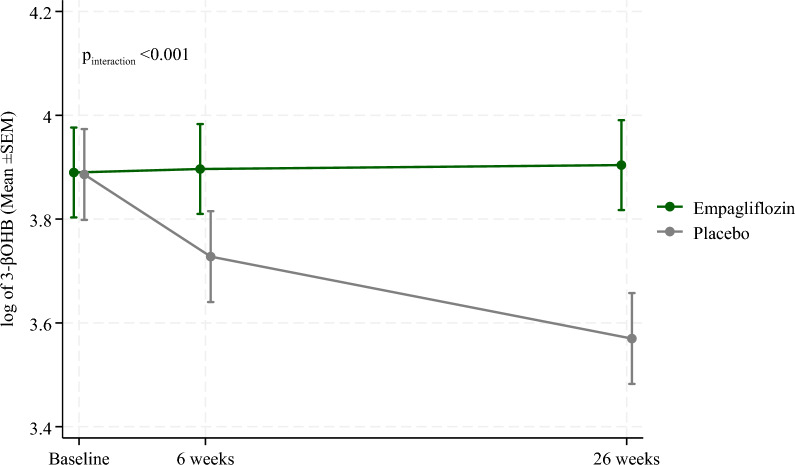
Mean ± SEM of log-transformed 3-βOHB (µmol/l) at each visit in Empagliflozin and Placebo groups. P_interaction_: P-value for interaction between treatment and visits

Table 2 shows that the mean ± SD level of 3-βOHB decreased slightly from 48.81 ± 2.85 µmol/l at baseline to 45.29 ± 2.24 µmol/l at 6 weeks and 42.04 ± 2.09 µmol/l at 26 weeks in the overall EMMY cohort. The mean 3-βOHB level slightly increased from 46.18 ± 3.00 µmol/l at baseline to 49.25 ± 2.21 µmol/l at 26 weeks in response to the Empagliflozin therapy, whereas it decreased from 51.65 ± 2.69 µmol/l at baseline to 35.77 ± 1.89 µmol/l at 26 weeks in the Placebo group. Figure 1, derived from the LMEM, supplements the data from Table 2 and shows that the Empagliflozin (p_interaction_ < 0.001) significantly improved or at least sustained 3-βOHB levels following AMI over 26 weeks of follow-up compared to the placebo group, in which 3-βOHB levels consistently dropped over visits.

**Table 2:** 3-βOHB levels at each visit

3-βOHB—µmol/l	Baseline	6 weeks	26 weeks
	(Mean ± SD)	(Mean ± SD)	(Mean ± SD)
All	48.81 ± 2.85	45.29 ± 2.24	42.04 ± 2.09
Empagliflozin	46.18 ± 3.00	48.76 ± 2.19	49.25 ± 2.21
Placebo	51.65 ± 2.69	41.99 ± 2.28	35.77 ± 1.89

### Association of 3-βOHB with cardiac markers

The LMEM analysis presented in Table 3 shows an insignificant positive association of both baseline and longitudinal 3-βOHB measurements with the change in NT-proBNP. Contrasting associations were observed for other cardiac markers with baseline and longitudinal 3-βOHB measurements. The baseline 3-βOHB and its tertiles were negatively associated with LVEF change at follow-up; however, the average change in 3-βOHB was positively associated with LVEF change over visits. Similarly, the baseline 3-βOHB and its tertiles were positively associated with LVESV and LVEDV over visits, whereas the average change in 3-βOHB over visits was negatively associated with LVESV and LVEDV change over visits. A similar albeit insignificant association was observed between the 3-βOHB and E/é ratio.

**Table 3:** Association of baseline and longitudinal 3-βOHB with cardiac markers with interaction effects by Empagliflozin treatment

Variables	Coefficient	95%CI	*P*-value	*P*-interaction
NT-proBNP-log				
3-βOHB-log (baseline)	0.047	− 0.001–0.095	0.057	0.649
3-βOHB tertiles (baseline)				
Tertile 2 vs. Tertile 1	0.090	− 0.035–0.215	0.158	0.273
Tertile 3 vs. Tertile 1	0.117	− 0.001–0.236	0.052	0.240
3-βOHB-log (longitudinal)	0.004	− 0.042–0.050	0.865	0.576
LVEF—%				
3-βOHB-log (baseline)	− 0.464	− 0.863–− 0.065	0.023	0.696
3-βOHB tertiles (baseline)				
Tertile 2 vs. Tertile 1	− 0.592	− 1.635–0.451	0.266	0.432
Tertile 3 vs. Tertile 1	− 1.319	− 2.299–0.339	0.008	0.326
3-βOHB-log (longitudinal)	0.595	0.156–1.035	0.008	0.306
LVESV—ml				
3-βOHB-log (baseline)	1.409	0.186–2.632	0.024	0.560
3-βOHB tertiles (baseline)				
Tertile 2 vs. Tertile 1	2.167	− 1.004–5.338	0.180	0.869
Tertile 3 vs. Tertile 1	3.482	0.524–6.436	0.021	0.938
3-βOHB-log (longitudinal)	− 2.099	− 3.443–− 0.755	0.002	0.980
LVEDV—ml				
3-βOHB-log (baseline)	0.640	− 1.170–− 2.449	0.488	0.477
3-βOHB tertiles (baseline)				
Tertile 2 vs. Tertile 1	2.742	− 1.920–7.405	0.249	0.647
Tertile 3 vs. Tertile 1	1.518	− 2.852–5.888	0.496	0.588
3-βOHB-log (longitudinal)	− 2.406	− 4.341–− 0.472	0.015	0.473
E/é ratio				
3-βOHB-log (baseline)	0.110	− 0.209 – 0.430	0.499	0.787
3-βOHB tertiles (baseline)				
Tertile 2 vs. Tertile 1	− 0.130	− 0.505–0.245	0.497	0.664
Tertile 3 vs. Tertile 1	0.290	− 0.060–0.641	0.104	0.915
3-βOHB-log (longitudinal)	− 0.117	− 0.298–0.065	0.207	0.266

P-interaction: P-value for interaction between 3-βOHB and treatment irrespective of visit. Coefficients are adjusted for age, sex, diabetes, and baseline values of each outcome

*NT-proBNP* N-terminal pro b-type natriuretic peptide**,** *LVEF* left ventricular ejection fraction, *LVESV* left ventricle end-systolic volume, *LVEDV* left ventricle end-diastolic volume, *E/é* left ventricular filling pressure

**Fig. 2:**
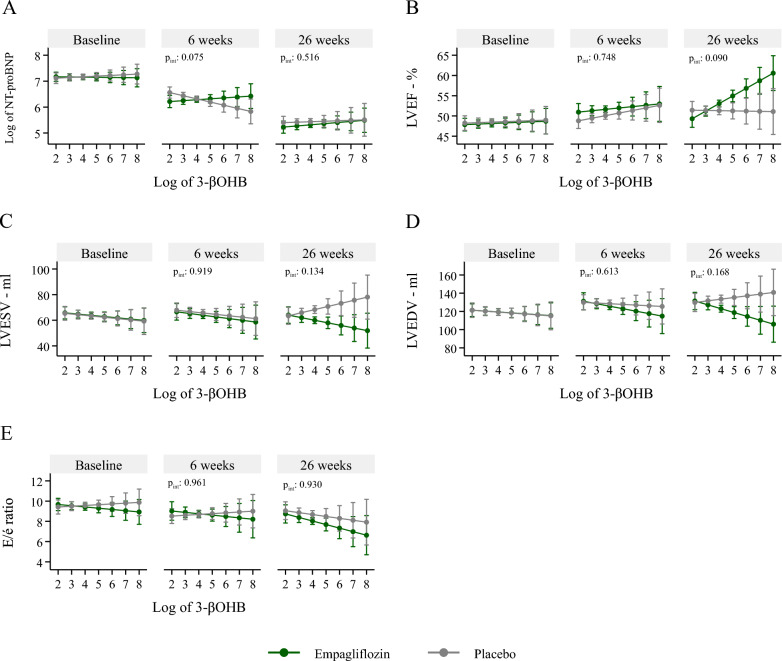
Interaction plots of 3-βOHB and cardiac markers at each visit. *NT-proBNP* N-terminal pro b-type natriuretic peptide**,** *LVEF* left ventricular ejection fraction, *LVESV* left ventricle end-systolic volume, *LVEDV* left ventricle end-diastolic volume, *E/é* left ventricular filling pressure

Table 3 and Fig. 2 present whether the associations between baseline and longitudinal measurements of 3-βOHB with cardiac markers were modified by Empagliflozin therapy. Although the results suggest a trend towards an interaction effect of Empagliflozin, none of the parameters reached statistical significance.

## Discussion

This post-hoc analysis of the EMMY trial investigated alterations in 3-βOHB in response to 26 weeks of Empagliflozin therapy compared to placebo in patients with AMI. In addition, the association of 3-βOHB levels measured within 72 h of AMI and follow-up visits with changes in cardiac biomarkers and functional as well as structural cardiac markers over visits was assessed in the entire EMMY cohort as well as its interaction with Empagliflozin therapy. The analysis showed a slight increase in 3-βOHB levels over 26 weeks in the Empagliflozin group compared to a consistent decline in 3-βOHB in the placebo group over time. The LMEM analysis of 3-βOHB with cardiac markers showed statistically insignificant associations of both baseline and longitudinal 3-βOHB measurements with NT-proBNP and E/é ratio. The baseline 3-βOHB was negatively associated with LVEF, whereas it was positively associated with LVESV and LVEDV over time. However, the association of longitudinal 3-βOHB measurements with LVEF change was positive, while it was negative for both LVESV and LVEDV. The interaction analysis demonstrated a potential albeit statistically insignificant interaction effect of Empagliflozin on the association between 3-βOHB, LVEF, LVESV, and LVEDV in the late post-MI phase.

Our analysis revealed high 3-βOHB levels within 72 h of PCI compared to follow-up visits. A previous study has also reported high levels of total ketone bodies, acetoacetate, acetone, and 3-βOHB within 24 h of STEMI [21]. Similarly, in a small study on patients with stable angina who underwent elective coronary angioplasty, a significant rise in ketone bodies following balloon occlusion was noted [25]. Myocardial ischemia and reperfusion are known to increase the bioavailability of ketone bodies and compromise their myocardial uptake and utilization [4]. In addition, ketone bodies may increase in the acute ischemic phase due to a direct stress response to MI and an increase in sympathetic drive, which in turn increases systematic catecholamines followed by lipolysis and free fatty acid production, thus resulting in ketosis and ketonemia [3].

Changes in ketone body concentration following myocardial ischemia and infarction are less explored in clinical settings, while animal studies from AMI models have not specifically focused on exploring alterations in ketone bodies over time. A recent study in patients with STEMI documented a persistent decline in total and individual circulatory ketone bodies over 4 months [21]. Consistent with this study, we observed a modest decline in 3-βOHB concentration from the ischemic/reperfusion phase to the post-ischemic phase in the overall EMMY cohort with a more pronounced decline in the placebo group. The declining trend in ketone bodies from ischemic to post-ischemic stages may indicate an increase in ketone body uptake and utilization and a progressive return of myocardium to normal energy metabolism.

In contrast to placebo, 3-βOHB concentrations increased slightly over 26 weeks of Empagliflozin therapy. Although such evidence is scarce in post-AMI clinical settings, our findings are not surprising and agree with previous experimental and preclinical data, which showed a significant role of SGLT2 inhibition in promoting ketogenesis, ketonemia, and uptake and utilization of ketone bodies, especially 3-βOHB, in the myocardium [17, 26, 27]. A recent systematic review of clinical and animal studies also supported that SGLT2i treatment significantly upregulated ketone metabolism and increased its plasma levels [28]. Our study fills this research gap in the clinical scenario by showing that SGLT2i play a significant role in increasing or at least sustaining ketone body levels in patients with recent MI. Interestingly, a higher elevation in 3-βOHB was noted from baseline to 6 weeks, while its levels remained constant between 6 and 26 weeks. This trend in 3-βOHB over time is somewhat discrepant with previously reported preclinical data [28], which might be explained by different animal models, study populations, and follow-up time considered in these studies. Despite this, our finding supports the therapeutic potential of ketone bodies in post-MI patients and accentuates the need for further research in exploring the long-term impact of SGLT2i on ketone body metabolism and the mediation of beneficial cardiometabolic effects.

Research assessing the relationship between ketone bodies and heart failure markers in post-MI settings is limited and has conflicting results. In our post-hoc analysis, both baseline and longitudinal measurements of 3-βOHB were not significantly associated with NT-proBNP. Consistent with our results, in a recent study of patients presenting with STEMI, no association was reported between acetone, acetoacetate, 3-βOHB, and NT-proBNP at baseline and 4-month follow-up [21]. In contrast, a study showed a positive correlation between total ketone bodies and B-type natriuretic peptide (BNP) in patients with existing CVD who underwent recent cardiac catheterization [29]. Given these conflicting findings, more research is recommended to understand the nature of the relationship between ketone bodies and heart failure biomarkers in different CVD populations.

The beneficial role of ketone bodies in improving cardiac function by increasing cardiac contractility, energy efficiency, and systemic vasodilation without substantially altering heart rate and blood pressure has been established in preclinical and experimental heart failure settings [1, 16, 30, 31]. However, evidence regarding the specific role of ketone bodies in ischemic heart conditions in both preclinical and clinical settings is still emerging. For instance, one-week treatment of Empagliflozin before induction of AMI in porcine models reduced MI size and preserved LVEF including other cardiovascular benefits [16]. Likewise, a recent clinical study revealed a positive association of ketone bodies after 24 h of STEMI with larger infarct size and lower ejection fraction [21]. In contrast, a study that enrolled patients with existing CVD who underwent recent cardiac catheterization found no direct correlation between total ketone body levels and various cardiac function parameters such as left ventricular end-diastolic pressure, LVESV index, and LVEDV index [29]. A unique aspect of our analysis is that it investigated the role of 3-βOHB during both ischemic/reperfusion and late phases of AMI, which revealed contrasting associations with functional cardiac markers. As such, increased 3-βOHB levels measured within 72 h of AMI were associated with lower LVEF and increased LVESV and LVEDV at follow-up. On the contrary, the change in 3-βOHB over 26 weeks had a positive impact on improving LVEF and decreasing end-systolic and end-diastolic volumes. These opposing results are not surprising and explain the difference in the role that ketone bodies might play during different post-MI phases. To elaborate, ketone body levels during ischemic/reperfusion phases directly correlate with the severity of ischemia, infarction, and stress response, and thus may serve as a potential biomarker for predicting future adverse CVD outcomes [21]. On the other hand, total ketones, especially 3-βOHB, are considered to be cardioprotective and their supplementation and infusion following MI was shown to improve cardiac function [16, 18, 22, 31, 32], which seems to be happening in our study participants as well owing to the empagliflozin therapy. Hence, our findings suggest the importance of investigating the longitudinal changes in ketone bodies in the post-MI context.

SGLT2i have been suggested to promote ketogenesis, ketonemia, and myocardial utilization of ketone bodies [4], while these drugs have also been shown to improve cardiac function and attenuate myocardial ischemia and reperfusion injury by acting on several mechanisms. In light of this evidence, we expected that Empagliflozin would significantly influence the correlations between 3-βOHB and cardiac markers. However, our interaction analysis produced insignificant results with an indication of some effect modification at 26 weeks. Despite this finding, we suggest more mechanistic research on investigating the role of SGLT2i in modifying the relationship between ketone bodies and cardiac markers in the late post-MI phase to gain more insights into the long-term clinical benefits of SGLT2i in this setting.

## Limitations

Ketone body levels were not available before AMI in the EMMY cohort, therefore, the magnitude of elevation in ketone bodies due to myocardial ischemia could not be evaluated. Also, it would have been more informative to collect sequential measurements of 3-βOHB during the initial hours and days of AMI, as rapid changes in its levels have been documented in the early post-MI phase. In addition, we could not detect a statistically significant modifying effect of Empagliflozin therapy on the association between 3-βOHB and cardiac markers possibly because the EMMY trial was not powered to investigate three-way interactions over time. Furthermore, we could not assess the mediatory role of 3-βOHB between Empagliflozin therapy and cardiac parameters because ketone body measurements were not available for all participants at all visits, which attenuated the true impact of Empagliflozin on outcomes observed in the entire EMMY cohort. Hence, the lack of a significant interaction effect of the Empagliflozin treatment despite a direct association of 3-βOHB with improvements in structural and functional cardiac markers might just be a matter of lack of statistical power. Therefore, we recommend further clinical research on this association within the ongoing EMPACT-MI trial (NCT04509674).

## Conclusions

This post-hoc analysis of the EMMY trial provides clinical evidence regarding the significant impact of SGLT2i on increasing circulatory 3-βOHB levels after AMI. In addition, elevated 3-βOHB levels following AMI were negatively associated with functional and structural cardiac markers at follow-up. At the same time, an increase in 3-βOHB over time significantly improved these markers, indicating the importance of time difference in understanding the role of ketones in metabolic and pathophysiological mechanisms of ischemic heart diseases and suggesting the clinical significance of longitudinal measurements of ketones. Last, the positive influence of 3-βOHB on cardiac markers was more obvious in the late post-MI stage and was evident in the SGLT2i group only, suggesting the moderation effect of SGLT2i in this scenario. However, the interaction effect did not reach statistical significance and thus we recommend more research in this direction.

## Data Availability

Data used in this post-hoc analysis will be shared upon reasonable request to the corresponding authors.

## Abbreviations

ACEI/ARB: Angiotensin-converting enzyme inhibitors/angiotensin receptor blockers
AMI: Acute myocardial infarction
ARNI: Angiotensin receptor/neprilysin inhibitor
ATP: Adenosine triphosphate
BMI: Body mass index
CAD: Coronary artery disease
CI: Confidence interval
CK: Creatine kinase
CK-MB: Creatine kinase muscle-brain
3-βOHB: β-Hydroxybutyrate
eGFR: Estimated glomerular filtration rate
EMMY: Empagliflozin in acute myocardial infarction
HDL: High-density lipoprotein
hsCRP: High-sensitivity C-reactive protein
IQR: Interquartile range
LDL: Low-density lipoprotein
LMEM: Linear mixed-effect model
MRA: Mineralocorticoid receptor antagonist
NSTEMI: Non-ST-elevation myocardial infarction
SEM: Standard error of mean
SGLT2i: Sodium-glucose co-transporter 2 inhibitors
LVEF: Left-ventricular ejection fraction
LVEDV: Left-ventricular end-diastolic volume
LVESV: Left-ventricular end-systolic volume
NT-proBNP: N-terminal pro b-type natriuretic peptide
E/é ratio: Left-ventricular filling pressure
